# Effect of sodium bicarbonate on cardiovascular outcome and mortality in patients with advanced chronic kidney disease

**DOI:** 10.3389/fphar.2023.1146668

**Published:** 2023-05-11

**Authors:** Ya-Lien Cheng, Shu-Chun Huang, Ming-Yun Ho, Yan-Rong Li, Chieh-Li Yen, Kuan-Hsing Chen, Wei-Chiao Sun, Pei-Yi Fan, Jung-Sheng Chen, Chihung Lin, Ching-Chung Hsiao

**Affiliations:** ^1^ Kidney Research Center and Department of Nephrology, Linkou Chang Gung Memorial Hospital, Taoyuan, Taiwan; ^2^ College of Medicine, Chang Gung University, Taoyuan, Taiwan; ^3^ Department of Physical Medicine and Rehabilitation, New Taipei Municipal Tucheng Hospital, Chang Gung Memorial Hospital, New Taipei, Taiwan; ^4^ Division of Cardiology, Department of Internal Medicine, Linkou Chang Gung Memorial Hospital, Taoyuan, Taiwan; ^5^ Division of Endocrinology and Metabolism, Department of Internal Medicine, Linkou Chang Gung Memorial Hospital, Taoyuan, Taiwan; ^6^ Department of Nephrology, New Taipei Municipal Tucheng Hospital, New Taipei, Taiwan; ^7^ Center for Artificial Intelligence in Medicine, Chang Gung Memorial Hospital at Linkou, Taoyuan, Taiwan

**Keywords:** sodium bicarbonate, advanced chronic kidney disease, cardiovascular outcome, mortality, dialysis

## Abstract

**Background:** Metabolic acidosis is a common complication in patients with chronic kidney disease (CKD). Oral sodium bicarbonate is often used to treat metabolic acidosis and prevent CKD progression. However, there is limited information about the effect of sodium bicarbonate on major adverse cardiovascular events (MACE) and mortality in patients with pre-dialysis advanced CKD.

**Method:** 25599 patients with CKD stage V between January 1, 2001 and December 31, 2019 were identified from the Chang Gung Research Database (CGRD), a multi-institutional electronic medical record database in Taiwan. The exposure was defined as receiving sodium bicarbonate or not. Baseline characteristics were balanced using propensity score weighting between two groups. Primary outcomes were dialysis initiation, all-cause mortality, and major adverse cardiovascular events (MACE) (myocardial infarction, heart failure, stroke). The risks of dialysis, MACE, and mortality were compared between two groups using Cox proportional hazards models. In addition, we performed analyzes using Fine and Gray sub-distribution hazard models that considered death as a competing risk.

**Result:** Among 25599 patients with CKD stage V, 5084 patients (19.9%) were sodium bicarbonate users while 20515 (80.1%) were sodium bicarbonate non-users. The groups had similar risk of dialysis initiation (hazard ratio (HR): 0.98, 95% confidence interval (CI): 0.95-1.02, *p* < 0.379). However, taking sodium bicarbonate was associated with a significantly lower risks of MACE (HR: 0.95, 95% CI 0.92–0.98, *p* < 0.001) and hospitalizations for acute pulmonary edema (HR: 0.92, 95% CI 0.88–0.96, *p* < 0.001) compared with non-users. The mortality risks were significantly lower in sodium bicarbonate users compared with sodium bicarbonate non-users (HR: 0.75, 95% CI 0.74–0.77, *p* < 0.001).

**Conclusion:** This cohort study revealed that in real world practice, use of sodium bicarbonate was associated with similar risk of dialysis compared with non-users among patients with advanced CKD stage V. Nonetheless, use of sodium bicarbonate was associated with significantly lower rate of MACE and mortality. Findings reinforce the benefits of sodium bicarbonate therapy in the expanding CKD population. Further prospective studies are needed to confirm these findings.

## Background

Metabolic acidosis (MA) is one of the common complications in patients with chronic kidney disease (CKD), especially when the glomerular filtration rate (GFR) is below 30 ml/min per 1.73 m^2^ (Kraut and Kurtz, 2005). The main pathogenesis of MA in CKD patients is the impaired ability to excrete nonvolatile acid, mainly ammonium, and reduced bicarbonate reabsorption and production. Failure to neutralize the net endogenous acid load will lead to acid retention (Kuczera, 2020). MA is generally defined as serum bicarbonate level falls below 22 mmol/L (Moranne et al., 2009). The GFR threshold for developing MA has not been clearly defined, but its prevalence increases as kidney function declines (Kuczera, 2020). It is estimated to be about 56% in CKD stage V patients (Kuczera, 2020).

Numerous observational studies have shown deleterious effects of MA on patients with CKD. Acidosis contributes to enhanced muscle catabolism (EUSTACE et al., 2004), inducing protein energy wasting (Ballmer et al., 1995), exacerbation of metabolic bone disease (Chen et al., 2015), more rapid progression of CKD (Menon et al., 2010), worse cardiovascular health and increased mortality (Dobre et al., 2013). To prevent these complications, The 2012 Kidney Disease Improving Global Outcomes (KDIGO) guidelines recommend alkali therapy for chronic metabolic acidosis to maintain venous bicarbonate levels between 24 and 26 mEq/L (Kidney Disease: Improving Global Outcomes KDIGO CKD Work Group, 2012). However, it is unclear whether these recommendations should apply unmodified to all CKD population. In addition, physicians may remain concerned about the potential harms of sodium-based alkali therapy in patients with CKD, including edema due to expansion of extracellular fluid, uncontrolled hypertension, and exacerbation of heart failure. MA is most common in pre-dialysis CKD stage V patients, who are more likely to require alkali therapy, but these patients are at the highest risk of developing side effects of sodium-based alkali therapy.

Two recent systematic reviews and meta-analyses of small trials showed that oral alkali supplementation modestly improved estimated GFR, but had an indeterminate effect on progression to dialysis and the effect on body weight, blood pressure and risk of edema was controversial (Hu et al., 2019; Navaneethan et al., 2019; Bi, 2020). The number of trials included in these systemic reviews was limited and these studies had small sample sizes, lacking long-term follow up for analyzing mortality and cardiovascular events. Moreover, the severity of CKD in these trials varied, mainly CKD stage III to IV. To our knowledge, only three intervention studies had examined the effect of oral sodium bicarbonate therapy in CKD stage V patients, but their results had shown different outcomes. In an open-label randomized study, Jeong et al. revealed the preservation of eGFR in CKD stage IV after sodium bicarbonate supplement for MA, but not in pre-dialysis CKD stage V. However, bicarbonate supplement improves nutritional indices in CKD stage V^13^. Iorio et al. (2019) reported that correction of MA with oral sodium bicarbonate in patients with CKD stage III-V significantly reduces the progression of stage V CKD to dialysis and improves patients’ survival irrespective of baseline renal function. Recently, data from the BiCARB study (Bi, 2020), a multicenter, double-blind, placebo-controlled trial which enrolled patients with aged > = 60 years with CKD stage IV or V, failed to observe any significant treatment effect of sodium bicarbonate on renal outcome including eGFR or risk of dialysis initiation. Moreover, oral sodium bicarbonate did not improve physical function but increase adverse events.

Alkali therapy in CKD may have beneficial effects, but the effects may differ depending on the stage of CKD. Few studies have focused on clinical outcomes and side effect of alkali therapy in pre-dialysis CKD stage V patients. Therefore, we conducted a retrospective cohort study employing Chang Gung Research Database (CGRD), a multi-institutional electronic medical record database in Taiwan, to determine whether administration of oral sodium bicarbonate can improve renal outcome or reduce risks of mortality and major adverse cardiovascular event (MACE) in patients with pre-dialysis CKD stage V.

## Methods

### Data source

Patient data were obtained from Chang Gung Research Database (CGRD). CGRD is a de-identified database derived from the electronic medical records (EMR) of Chang Gung Memorial Hospital (CGMH), Taiwan’s largest healthcare provider, which includes four tertiary medical centers and three teaching hospitals across different regions of Taiwan, with a total of 10,050 beds and approximately 280,000 admissions annually (Shao et al., 2019), accounting for approximately 10% of all medical services in Taiwan. Before the data are released to researchers, any information in the CGRD that could identify any particular patient or healthcare provider is scrambled and encrypted to ensure privacy. The need for patient consent was waived and the study protocol was approved by the Institutional Review Board of Chang Gung Medical Foundation, Taiwan (IRB No: 202201897B0).

### Patient selection and study design

**FIGURE 1:**
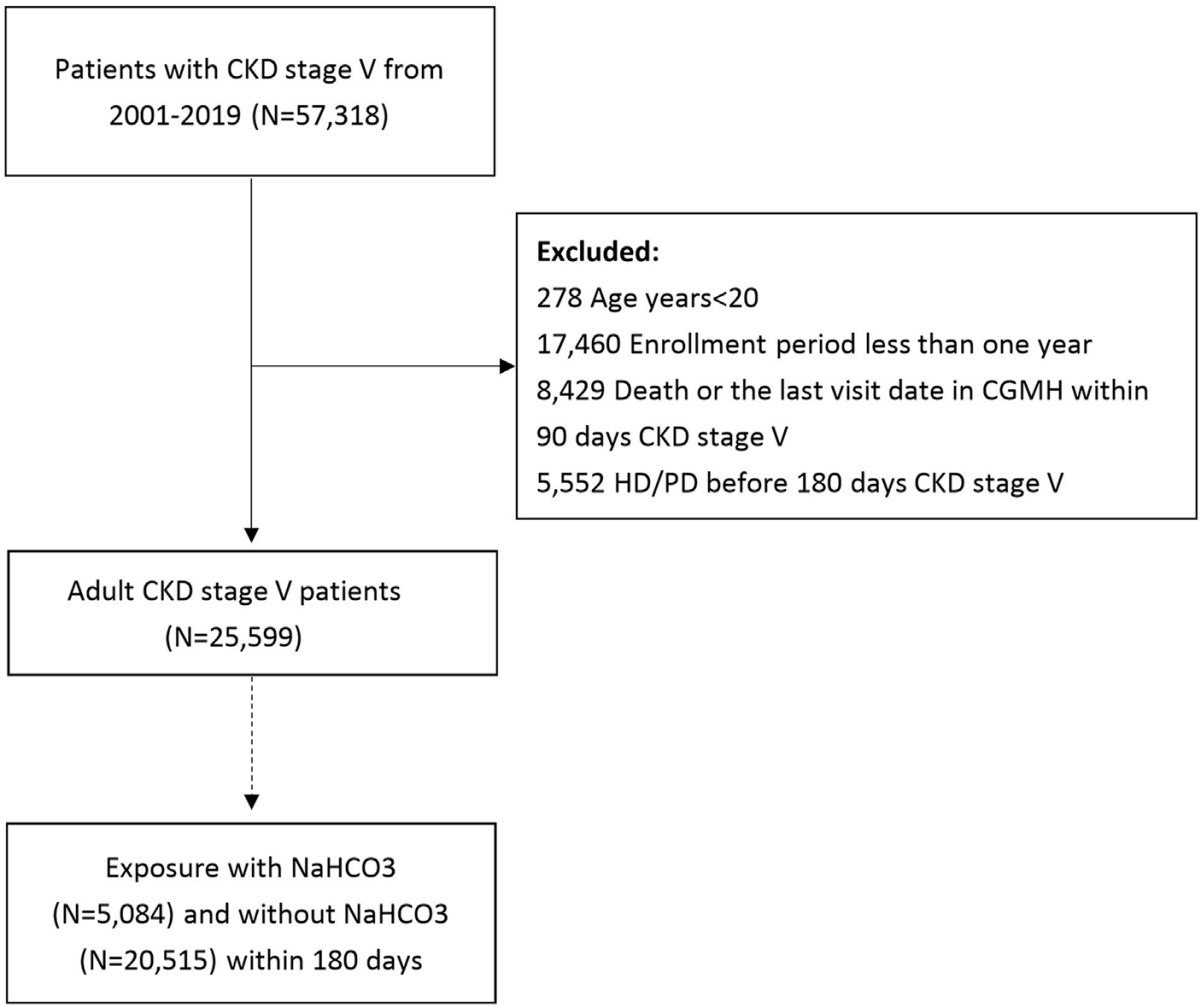
Selection process of study cohort.

We designed a retrospective cohort study to evaluate the effect of oral sodium bicarbonate on mortality and cardiovascular outcome in patients with advanced CKD. As illustrated in Figure 1, patients diagnosed with CKD stage V, which is defined as of patients with CKD stage V diagnosis (ICD9:585.5; ICD10:N185) and estimated GFR less than 15 ml/min/1.73 m (Kuczera, 2020) using CKD-EPI equation (Levey and Stevens, 2010) between January 1, 2001, and December 31, 2019 were identified by searching electronic medical records from the CGRD. The index date was defined as 180 days after the date of patient diagnosed with CKD stage V. Patient’s age <20 (N = 278), enrolled for less than 1 year (N = 17460), and death or date of last visit within 90 days of CKD stage V (N = 8429) were excluded from this study. To avoid participants with acute kidney injury receiving temporary hemodialysis, we excluded patients receiving hemodialysis or peritoneal dialysis within 180 days before the index date (N = 5552). Finally, 25599 patients with CKD stage V were included in the study cohort. We used landmark analysis with exposure windows of 180days to define oral sodium bicarbonate exposure (Dafni, 2011; Ravi et al., 2013). Sodium bicarbonate exposure was defined as CKD stage V patients who had received the first prescription for sodium bicarbonate within 180days of index date. Among 25599 patients, there were 5084 sodium bicarbonate users and 20515 sodium bicarbonate non-users.

### Covariates and study outcomes

Baseline demographic and clinical characteristics were identified using International Classification of Diseases, 9th Revision, Clinical Modification (ICD-9-CM) codes prior to 2016 or ICD-10-CM codes later. Baseline characteristics in our study included age, sex, Charlson Comorbidity Index (CCI), comorbidities, medications, laboratory values, and follow-up years. The comorbidities in question were hypertension, diabetes mellitus (DM), hyperlipidemia, stroke, coronary artery disease (CAD), myocardial infarction (MI), heart failure, peripheral artery disease, atrial fibrillation, liver cirrhosis, chronic obstructive pulmonary disease (COPD), hepatitis B virus/hepatitis C virus (HBV/HCV), and dementia. Comorbidities were identified based on the presence of more than 2 outpatient visits or at least one inpatient admission within the year preceding the index date. Most of the diagnostic codes used for these comorbidities have been validated in previous national database studies (Wu et al., 2014; Hsieh et al., 2015). Baseline medications were identified based on filling prescriptions at least twice or refilling a prescription for a chronic illness at least once within the year prior to the index date. Baseline laboratory results including the estimated glomerular filtration rate (eGFR), hemoglobin, sodium, potassium, calcium and albumin levels were obtained using the most recent record within 3 months preceding the index date.

The key outcomes were all-cause mortality, dialysis (hemodialysis or peritoneal dialysis), MACE (myocardial infarction, heart failure and stroke) and acute pulmonary edema. The diagnoses of dialysis, MACE and acute pulmonary edema were based on the principal diagnosis in the emergency department or hospitalization. The

Follow-up duration was from the index date until the first occurrence of death, MACE, dialysis, or the end of the follow-up period (December 31, 2019), whichever came first.

### Statistical analysis

The baseline characteristics showed substantial differences between the study groups (NaHCO3 *vs*. non-NaHCO3), which may induce selection bias. Thus, we used

Inverse probability of treatment weighting (IPTW) based on propensity score to balance the baseline differences between the two groups (Xu et al., 2010; Austin, 2014). Propensity score was defined as the probability of a patient receving NaHCO3 treatment, which was calculated by the logistic regression model using age, sex, follow-years, Charlson comorbidity index, comorbidity, medication and laboratory values.

The balance between groups before and after IPTW was assessed using the absolute value of standardized mean differences, and absolute value less than 0.1 indicates non-substantial difference between groups (Austin, 2011). The risk of mortality between the NaHCO3 and non-NaHCO3 groups was compared using Cox proportional hazard model. For other time to event outcomes, we used the Fine and Gray sub-distributional hazards model, which considered all-cause mortality as a competing risk.

Subgroups analyses were conducted on MACE and mortality stratified by age groups, sex, dialysis, diabetes, heart failure, myocardial infarction, stroke, and atrial fibrillation. Analyses were performed using SAS Version 9.4 (SAS Institute, Cary, NC). A two-sided *p*-value of <0.05 was considered significant.

## Results

### Subject characteristics

A total of 25599 CKD stage V patients who met the inclusion criteria between January 1, 2001, and December 31, 2019 were extracted from CGRD and divided into NaHCO3 users (n = 5084) and NaHCO3 non-users (n = 20515) groups according to their NaHCO3 exposure. The baseline characteristics of each group are listed in Table 1. The mean age (years) of NaHCO3 users was 67.5.1 ± 13.7 and that of NaHCO3 non-users was 66.3 ± 13.8. Compared with NaHCO3 non-users, NaHCO3 users had shorter follow-up, lower baseline eGFR, calcium, and albumin level. NaHCO3 users are more likely to receive medications including thiazide, oral hypoglycemic agents, and statin than NaHCO3 non-users. After the PS weighting, all ASMD values were less than 0.1, suggestive of well-balanced baseline demographic and clinical characteristics between groups.

**TABLE 1:** Demographic characteristic of study population before and after propensity scores matching.

	Before PS weighting					After PS weighting		
Variables	NaHCO3 (n = 5,084)		Non-NaHCO3 (n = 20,515)		ASMD*	NaHCO3 (n = 5,084)	Non-NaHCO3 (n = 20,515)	ASMD*
Male, n (%)	2,649	(52.1)	10,057	(49.0)	0.06	49.40%	49.6%	<.01
Age years, mean ± SD	67.5	±13.7	66.3	±13.8	0.09	66.1 ± 31.3	66.5 ± 15.4	0.02
Age groups, n (%)								
20–44	310	(6.1)	1,408	(6.9)	0.11	7.0%	6.7%	<.01
45–64	1,652	(32.5)	7,185	(35.0)		34.7%	34.5%	
65–74	1,379	(27.1)	5,673	(27.7)		27.7%	27.6%	
75+	1,743	(34.3)	6,249	(30.5)		30.5%	31.2%	
CCI, mean ± SD	3.9	±2.9	3.5	±2.8	0.12	3.6 ± 6.4	3.6 ± 3.1	0.01
CCI, n (%)								
0	491	(9.7)	2,638	(12.9)	0.12	12.6%	12.2%	0.04
1	621	(12.2)	2,872	(14.0)		13.5%	13.6%	
2+	3,972	(78.1)	15,005	(73.1)		74.0%	74.1%	
Follow years, mean ± SD	3.2	±3.1	4.4	±3.6	0.37	4.2 ± 8.5	4.2 ± 3.9	<.01
Comorbidities, n (%)								
Diabetes mellitus	3,033	(59.7)	11,294	(55.1)	0.09	56.0%	56.0%	<.01
Hypertension	4,133	(81.3)	15,984	(77.9)	0.08	78.4%	78.6%	0.01
Hyperlipidemia	1,521	(29.9)	6,641	(32.4)	0.05	32.2%	31.9%	0.01
Stroke	1,216	(23.9)	4,812	(23.5)	0.01	22.5%	23.5%	0.02
CAD	1,358	(26.7)	5,550	(27.1)	0.01	26.7%	27.0%	0.01
Myocardial infarction	630	(12.4)	2,252	(11.0)	0.04	11.0%	11.3%	0.01
Heart failure	1,511	(29.7)	5,419	(26.4)	0.07	26.7%	27.1%	0.01
Peripheral artery disease	504	(9.9)	2,078	(10.1)	0.01	9.9%	10.1%	0.01
Atrial fibrillation	514	(10.1)	1,756	(8.6)	0.05	8.4%	8.8%	0.01
Liver cirrhosis	747	(14.7)	2,906	(14.2)	0.02	14.6%	14.3%	0.01
COPD	956	(18.8)	3,349	(16.3)	0.07	16.7%	16.8%	<.01
HBV	89	(1.8)	325	(1.6)	0.01	1.6%	1.6%	<.01
HCV	341	(6.7)	1,327	(6.5)	0.01	6.3%	6.5%	0.01
Dementia	366	(7.2)	1,445	(7.0)	0.01	6.8%	7.1%	0.01
Medication, n (%)								
Aspirin/Clopidogrel	2,628	(51.7)	9,965	(48.6)	0.06	49.1%	49.2%	<.01
ACEI/ARB	4,429	(87.1)	17,297	(84.3)	0.08	85.4%	84.9%	0.02
Beta-blocker	4,277	(84.1)	16,412	(80.0)	0.11	81.2%	80.9%	0.01
CCB	913	(18.0)	2,711	(13.2)	0.13	14.4%	14.2%	<.01
K-sparing diuretics	553	(10.9)	2,045	(10.0)	0.03	10.2%	10.1%	<.01
Thiazide	4,290	(84.4)	14,927	(72.8)	0.29	75.1%	75.1%	<.01
Loop diuretics	2,603	(51.2)	9,409	(45.9)	0.11	47.4%	47.0%	0.01
OHA	3,533	(69.5)	11,341	(55.3)	0.3	57.7%	58.1%	0.01
Insulin	2,090	(41.1)	8,660	(42.2)	0.02	42.2%	42.1%	<.01
Statin	1,050	(20.7)	2,632	(12.8)	0.21	14.6%	14.4%	0.01
Lab (baseline)								
eGFR	9.6	±3.7	10.5	±3.6	0.24	10.4 ± 8.0	10.3 ± 4.1	0.01
Hb	9.7	±1.6	10	±1.7	0.18	9.9 ± 3.7	9.9 ± 1.8	<.01
K	4.4	±1.0	4.4	±0.7	0.05	4.4 ± 1.8	4.4 ± 1.1	0.01
P	4.6	±1.5	4.5	±1.2	0.11	4.5 ± 3.1	4.5 ± 1.4	<.01
Ca	8.6	±0.8	8.8	±0.8	0.24	8.7 ± 1.9	8.7 ± 0.9	<.01
Albumin	3.5	±0.6	3.7	±0.6	0.26	3.7 ± 1.4	3.7 ± 0.6	0.01

CAD, Coronary artery disease; COPD, Chronic obstructive pulmonary disease; HBV, Hepatitis B; HCV, Hepatitis C; ACEI, Angiotensin-converting enzyme inhibitors; ARB, Angiotensin receptor blockers; CCB, Calcium channel blocker; OHA, Oral hypoglycemic agent; ASMD, Absolute value of standardized mean differences.

*ASMD, greater than 0.1 was considered as a sign of imbalance.

### Effect of oral sodium bicarbonate on dialysis and MACE in patients with advanced CKD

Table 2 shows the risk of dialysis, MACE, and mortality in NaHCO3 users compared to NaHCO3 non-users after IPTW. The Cox proportional hazards model with competing risk analysis showed no difference in dialysis risks (HR: 0.98, 95% CI 0.95–1.02, *p* = 0.379) between the two groups. Those using NaHCO3 demonstrated a lower risk of MACE (HR: 0.95, 95% CI 0.86–0.97, *p* < 0.001) compared to NaHCO3 non-users. Regarding the components of MACE, NaHCO3 users had lower risk of myocardial infarction (HR: 0.91, 95% CI 0.86–0.97, *p* = 0.002) and stroke (HR: 0.92, 95% CI 0.88–0.96, *p* < 0.001) than NaHCO3 non-users.

**TABLE 2:** Frequency and Propensity Scores Weighting (PSW) Hazard Ratio Hazard Ratios with/without Competing Risk for Interested Outcomes by NaHCO3 Exposure.

Outcome	NaHCO3		Non-NaHCO3		P^a^	PSW Hazard Ratio (95% CI)	p	PSW Hazard Ratio with competing risk (95% CI)	p
	Events	(%)	Events	(%)					
Dialysis	1,171	(23.0)	5,098	(24.9)	0.007	0.94 (0.91 to 0.98)	0.001	0.98 (0.95 to 1.02)	0.379
MACE	1,793	(35.3)	7,415	(36.1)	0.244	0.91 (0.88 to 0.93)	<.001	0.95 (0.92 to 0.98)	<.001
MI	438	(8.6)	1,833	(8.9)	0.473	0.85 (0.80 to 0.90)	<.001	0.91 (0.86 to 0.97)	0.002
HF	1,111	(21.9)	4,386	(21.4)	0.462	0.92 (0.89 to 0.96)	<.001	0.97 (0.94 to 1.01)	0.158
Stroke	756	(14.9)	3,515	(17.1)	<.001	0.88 (0.84 to 0.92)	<.001	0.92 (0.88 to 0.96)	<.001
Acute pulmonary edema	704	(13.9)	3,050	(14.9)	0.066	0.88 (0.84 to 0.92)	<.001	0.92 (0.88 to 0.96)	<.001
Death	2,539	(49.9)	11,683	(57.0)	<.001	0.75 (0.74 to 0.77)	<.001	N/A	
Dialysis prior to death	566	(11.1)	2,988	(14.6)	<.001	0.66 (0.63 to 0.69)	<.001	N/A	
Death without Dialysis	1,973	(38.8)	8,695	(42.4)	<.001	0.79 (0.76 to 0.81)	<.001	N/A	

PSW, propensity score weighting.

^a^ *p* values was derived from Pearson’s chi-square tests to test the difference between NaHCO3 users and Non-NaHCO3, users by different outcomes.

Effect of oral sodium bicarbonate on acute pulmonary edema and mortality in patients with advanced CKD.

The Cox proportional hazards model with competing risk analysis revealed that NaHCO3 users had a lower risk of acute pulmonary edema (HR: 0.92, 95% CI 0.88–0.96, *p* < 0.001) than NaHCO3 non-users. Regardless of patients entering dialysis prior to death or died without dialysis, NaHCO3 users had a lower risk of mortality (HR: 0.75, 95% CI 0.74–0.77, *p* < 0.001) compared with NaHCO3 non-users.

#### Subgroup analysis

To verify whether clinical conditions modified the association between the use of NaHCO3 and primary outcomes, we performed subgroup analyses for outcomes of MACE and mortality (Tables 3, 4). The results were generally consistent in favor of NaHCO3 users except for atrial fibrillation and stroke in MACE, and atrial fibrillation in mortality. Patients without atrial fibrillation and stroke benefited more from the protective effect of NaHCO3 in MACE.

**TABLE 3:** Event numbers and hazard ratios for MACE between NaHCO3 user and Non-NaHCO3 usera.

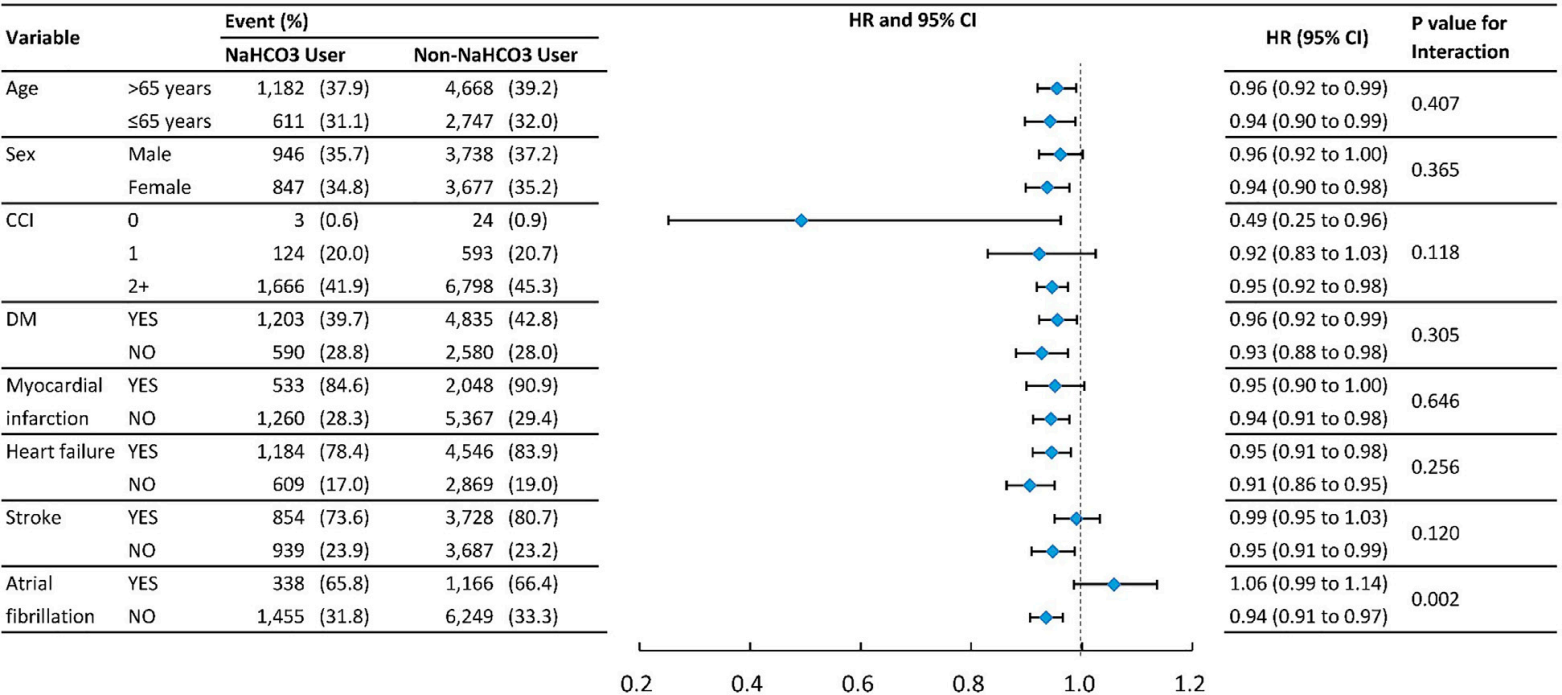

^a^ The hazard ratios for sub-groups were derived from the cox model and weighting by the propensity score with the competing risk for death.

**TABLE 4:** Event numbers and hazard ratios for mortality between NaHCO3 user and Non-NaHCO3 usera.

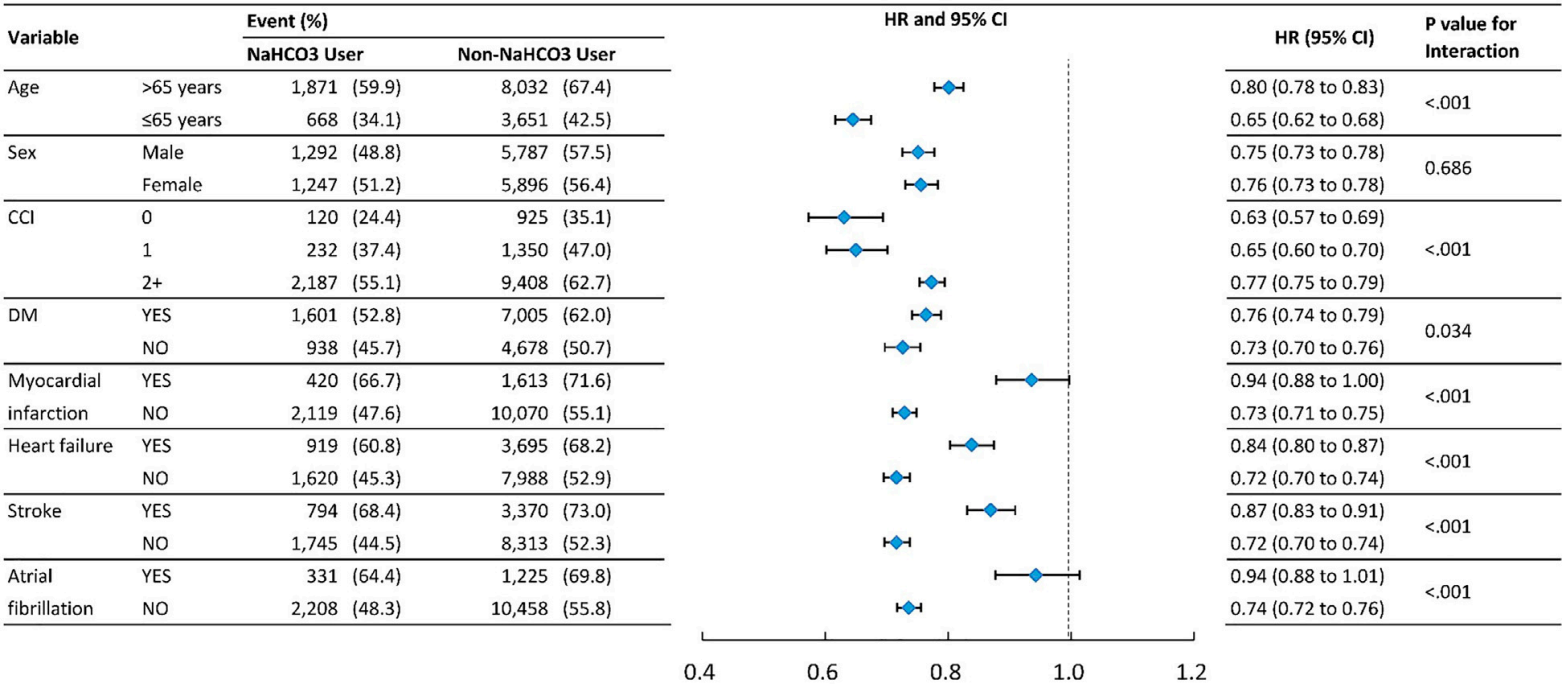

^a^ The hazard ratios for sub-groups were derived from the cox model and weighting by the propensity score.

## Discussion

The key findings of this retrospective multi-institutional observational study are: 1) use of oral sodium bicarbonate in patients with advanced CKD reduces the risk of MACE, mainly in reducing the events of myocardial infarction and stroke; 2) patients with advanced CKD treated with sodium bicarbonate have a lower risk of death and acute pulmonary edema during clinical follow-up compared with sodium bicarbonate non-users; and 3) nevertheless, sodium bicarbonate treatment fails to decrease the risk of dialysis in patients with pre-dialysis CKD stage V. An intriguing result of this research provides real-world outcome and additional clinical evidence for a better understanding of the pathophysiology and long-term complication in the advanced CKD population.

## Effect of sodium bicarbonate on renal progression in advanced CKD

Previous studies proposed possible mechanisms linking MA to rapid progression of CKD, which included activation of alternative complement cascade driven by compensatory increases in renal ammonia production in residual nephrons (Nath et al., 1985), triggering the production of endothelin and proinflammatory cytokines (Wesson et al., 2007; Phisitkul et al., 2008), and stimulation of the renin-angiotensin system (Wesson and Simoni, 2010; Ng et al., 2011; Wesson et al., 2015), each of which promotes acute acid excretion but chronically contributes to tubulointerstitial damage and fibrosis. An observational cohort study with participants with CKD stage II-IV showed that low serum bicarbonate level was an independent risk factor for kidney disease progression, however, it is also important to note that this association was stronger for participants with eGFR above 45 ml/min/1.73 m^2^ (Paul Chubb et al., 2016). A number of potential protective mechanisms of sodium bicarbonate supplementation have been identified (Mannon and O'Connor, 2020). One possible mediating mechanism is that alkali corrects the renal compensatory response to an acidic milieu, including lowering interstitial NH_4_^+^ concentration, resulting in decreased complement activation, reduced interstitial acidosis, and thus reduced local production of endothelin-1 and angiotensin II, as well as reducing H^+^ secretion and thereby preventing tubular cast formation. Another extrarenal regulation mechanism is to reduce renal inflammation by activating cholinergic anti-inflammatory pathway (Ray et al., 2018) and to improve glycemic control and metabolic status by correcting acidosis (Bellasi et al., 2016). Alkali supplementation may provide protection on renal long-term outcomes and may also influence other disease states in CKD population through two regulatory mechanisms. However, not all interventional studies enrolling advanced CKD population have established the benefit of using sodium bicarbonate in delaying renal disease progression to ESRD (de Brito-Ashurst et al., 2009; Jeong et al., 2014; Iorio et al., 2019; Bi, 2020). In this study, we were unable to demonstrate that oral sodium bicarbonate successfully delayed the progression of advanced CKD to dialysis in pre-dialysis CKD stage V patients. This may be explained by the competing risk of death in this vulnerable population, short treatment duration from CKD stage V to dialysis and complex uremic environment causing rapid renal progression. These results may not necessarily conclude that sodium bicarbonate is not effective in halting renal progression in this population. Further studies are warranted to confirm these findings.

## Effect of sodium bicarbonate on MACE in advanced CKD

To the best of our knowledge, this is the first cohort study to evaluate the effect of oral sodium bicarbonate therapy on MACE in the advanced CKD population. The association of serum bicarbonate with atherosclerotic cardiovascular disease and heart failure has been poorly understood, and the results are inconsistent across different study population. The Chronic Renal Insufficiency Cohort (CRIC) study investigating patients with CKD stages II–IV illustrated an increased incidence of heart failure with serum bicarbonate levels above 24–26 mEq/L, but there is no significant association with atherosclerotic events, including coronary artery disease. (Dobre et al., 2013; Dobre et al., 2015). However, it is important to note that the CRIC study excluded patients with New York Heart Association class III and IV heart failure. In the cohort of all patients with type 2 DM with varying degrees of kidney function, serum bicarbonate level was independently and inversely associated with incident coronary heart disease, but not with heart failure (Paul Chubb et al., 2016). The data from patients with diabetic nephropathy who enrolled in the Reduction of End points in Non-insulin-dependent diabetes with the Angiotensin II Antagonist Losartan (RENAAL) trial or the Irbesartan Diabetic Nephropathy Trial (IDNT) showed no relationship between bicarbonate level and cardiovascular events, heart failure (Schutte et al., 2015). The RENAAL/IDNT study also exclude patients who had a history of heart failure. In contrast, another current large analysis of hypertensive individuals without diabetes showed that serum bicarbonate level less than 22 mEq/L increase the risk of fatal and nonfatal cardiovascular events (Dobre et al., 2020).

The detailed mediating mechanisms linking serum bicarbonate to heart failure and the discrepancy across different cohort phenotypes require advanced research. Our study demonstrated that the use of sodium bicarbonate dose not increase the risk of heart failure, thereby reducing the doubts and concerns about the clinical use of this drug in pre-dialysis CKD stage V patients. The results echo previous experiences that sodium bicarbonate administration did not significantly affect blood pressure, edema, or weight gain, and it was well tolerated (de Brito-Ashurst et al., 2009; Iorio et al., 2019; Dubey et al., 2020).

Moreover, for the first time, we found that oral sodium bicarbonate therapy reduce the risk of MACE, including myocardial infarction and stroke in CKD population. Cardiovascular events are the leading cause of death in patients with chronic kidney disease. In addition to traditional cardiovascular risk factors such as hypertension and dyslipidemia, metabolic acidosis has been postulated as one of the potential mechanisms for progression of CKD. Acidosis stimulates the expression of multiple inflammatory genes and upregulates endothelial cell adhesion in endothelial cells (Chen et al., 2011; Dong et al., 2013; Dobre et al., 2020). This leads to recruitment and activation of leukocytes and plasma leakage, which in turn cause tissue damage. The persisting low-grade systemic inflammation impairs endothelial function and predisposes to accelerated atherosclerosis (Recio-Mayoral et al., 2011). Thus, the link between metabolic acidosis and atherosclerotic heart disease may be mediated through endothelial inflammatory processes (Dobre et al., 2020). Furthermore, acidosis is associated with the activation of the renin-angiotensin-aldosterone system, and elevated aldosterone levels may contribute to increase the incidence of cardiovascular and atherosclerotic disease (Hillege et al., 2000). Recently, Kendrick et al. performed a pilot randomized crossover study showing that a 6-week supply of sodium bicarbonate has favorable effects on vascular endothelial function, as assessed by flow-mediated dilation, in patients with stage IIIb and IV CKD (Kendrick, 2018). Although the research have not been entirely conclusive, it is plausible that taking sodium bicarbonate to correct acidosis and suppress chronic inflammation reduces the risk of MACE in advanced CKD population.

### Effect of sodium bicarbonate on mortality in advanced CKD

Serum bicarbonate tends to display a U-shaped relationship with mortality from prior observational studies in moderate and advanced CKD (Kovesdy et al., 2009). Patients with serum bicarbonate level <22 mmol/L and > 29 mmol/L were at increased risk of death (Navaneethan et al., 2011). Our findings demonstrate that sodium bicarbonate supplementation reduces risk of death in pre-dialysis CKD stage V patients. This result is consistent with a recent randomized clinical trial study, the UBI study, which reported a survival benefit in patients with CKD stages III-V, who achieve a target serum bicarbonate level of 24–28 mmol/L by taking sodium bicarbonate (Iorio et al., 2019). We also observed a protective effect of sodium bicarbonate in reducing the risk of death both prior to and after entering dialysis. Although the mechanisms are uncertain, this may be due in part to the protective effect of alkali therapy on reducing the risk of MACE. Moreover, malnutrition could be reversed with sodium bicarbonate treatment, which may also partially explain our finding of reduced mortality. Approximately 40% of patients at the start of dialysis suffer from protein-energy wasting, and malnutrition is thought to be one of the major risks of increased morbidity and death in CKD population (Ikizler et al., 1995). Extensive data from short-term metabolic studies indicate that chronic metabolic acidosis in CKD results in decreased protein synthesis, enhanced muscles proteolysis, endocrine abnormalities (e.g., insulin resistance), ultimately the development of protein-energy wasting (Ballmer et al., 1995; EUSTACE et al., 2004). And available interventional evidence confirms oral bicarbonate supplementation increases serum albumin and has positive nutritional benefits among patients with CKD and dialysis patients (de Brito-Ashurst et al., 2009; Jeong et al., 2014; Dubey et al., 2020). Attenuating malnutrition and inflammation by treating metabolic acidosis may contribute to reduce mortality. One potential clinical concern issue is that excess administration of exogenous alkali therapy may result in metabolic alkalosis and associated side effects. However, according to previous studies, serum bicarbonate level did not exceed normal values when administrated for 1 year at a daily dose of oral sodium bicarbonate at 1 mEq/kg body weight/d (5.9 g/d for 70 kg body weight) in CKD stage IV patients with MA (Goraya et al., 2013). Therefore, sodium bicarbonate at doses (2–3 g/d), which are primarily used in patients with CKD, are unlikely to cause this complication (Goraya and Wesson, 2017).

This study has several limitations that should be acknowledged. First, to assess the effects of sodium bicarbonate in CKD stage V, we defined sodium bicarbonate exposure as a prescription within 180 days from the date of a diagnosis of CKD stage V. Patients who were prescribed sodium bicarbonate during early CKD were excluded. The influence of different drug regimens and dosages was beyond the scope of this study. Full prescription and refill information was not available as patients are not obliged to take their medications at our health care facilities. There was also incomplete information on medication adherence. Second, even though PSW analysis was adopted to minimize relevant confounders that we could identify, the observational nature of this study made complete elimination of other residual biases impossible and may have inherent limitations. Third, our study is a multi-center study which did not account for site specific environmental factors as urban *versus* rural settings, academic hospitals *versus* community hospitals, the reproducibility and generalizability of this report will need further validation.

In conclusion, our study revealed that oral sodium bicarbonate therapy might reduce the risk of MACE and mortality in patients with pre-dialysis CKD stage V without increasing the incidence of heart failure and acute pulmonary edema. This inexpensive and simple strategy is consistent with current renal care recommendations and has the potential to yield significant economic, quality of life and clinical outcome benefits in the expanding CKD population (Fine and Gray, 1999).

## Data Availability

The raw data supporting the conclusion of this article will be made available by the authors, without undue reservation.
